# Outcome of enzyme replacement therapy for hematological and visceral manifestations in children with acid sphingomyelinase deficiency: a single center experience in upper Egypt

**DOI:** 10.1186/s40348-025-00199-9

**Published:** 2025-08-14

**Authors:** Mervat A. M. Youssef, Esraa Hefzy Shaker, Nahed A M Saleh

**Affiliations:** 1 Pediatric Hematology Unit, Faculty of Medicine, Children’s Hospital, Assiut University, Assiut, Egypt; 2 Mediclinic Middle East, Dubai, UAE

**Keywords:** ASMD, Pediatric, Thrombocytopenia, Anemia, Enzyme replacement therapy, Olipudase alfa

## Abstract

**Background:**

Thrombocytopenia is the most common hematologic manifestation of acid sphingomyelinase deficiency (ASMD). The introduction of enzyme replacement therapy (ERT) represents significant progress in the treatment landscape of this disorder. This study presents the largest pediatric case series of ASMD to date, providing valuable insights into the real-world application of ERT in affected children.

**Methods:**

Ten children with ASMD (five with type B and five with type A/B) received ERT for one year. Growth parameters, complete blood counts, abdominal ultrasonography, liver function tests, lipid profiles, and neurological assessments were conducted at baseline and subsequently every three months. In addition, chest high-resolution computed tomography (HRCT) and dual-energy X-ray absorptiometry (DXA) were performed at baseline and repeated after one year.

**Results:**

No serious infusion-related reactions (IAR) were recorded. However, one patient developed a mild urticarial rash, while another experienced pyrexia. Anemia was present in all children at baseline. A significant increase in hemoglobin levels starting at week 12 (*p* = 0.02) with peak levels observed at week 50. Thrombocytopenia was present in 60% of patients at baseline. Platelet counts did not show a significant change at week 12 (*p* = 0.3), but a significant increase was observed after 24 weeks (*p* = 0.0196), and counts peaked at week 50 (*p* = 0.0057). There was a significant reduction in liver and spleen sizes, as well as lipid profile parameters. In addition, gradual improvements were observed in interstitial lung disease scores and bone mineral densities throughout the study course.

**Conclusion:**

Our findings indicate that olipudase alfa provides significant benefits in key hematological and visceral clinical outcomes in pediatric patients with ASMD.

## Introduction

Acid sphingomyelinase deficiency (ASMD) is a rare lysosomal storage disorder with an autosomal recessive inheritance pattern, caused by mutations in the SMPD1 gene [1]. These mutations lead to a deficiency of the acid sphingomyelinase enzyme, resulting in the accumulation of sphingomyelin in multiple tissues throughout the body. This accumulation contributes to the diverse phenotypic manifestations observed in patients with ASMD.

Historically, ASMD was classified as part of Niemann–Pick disease (NPD) and subdivided into two subtypes: Niemann-Pick disease type A (NPD A) and type B (NPD B). Both subtypes result from a deficiency of the human sphingomyelin-cleaving enzyme [2]. Subsequent clinical and biochemical investigations identified a specific subtype, referred to as Niemann-Pick type C (NPC), which is caused by a defect in cholesterol trafficking, a mechanism different from the sphingomyelin-cleaving enzyme deficiency [3]. Currently, NPDs are classified into two distinct groups: ASMD (including both types A and B) and NPC [4]. Some patients with ASMD exhibit an intermediate phenotype, known as the chronic neurovisceral form (ASMD A/B or intermediate ASMD). This form is characterized by significant somatic manifestations with progressive neurological symptoms [5]. Due to the wide range of clinical severity, life expectancy in ASMD can vary widely, ranging from premature death to extended survival with significant health challenges.

The clinical presentations of ASMD types A and B differ significantly, which influences patient outcomes. ASMD type A is considered the most severe form, typically presenting in early infancy and characterized by a near-complete absence of residual ASM activity. This results in rapidly progressive systemic manifestations, particularly hepatosplenomegaly, along with severe central nervous system deterioration. Unfortunately, mortality is inevitable in ASMD type A, where most patients die within the first three years of life [6].

ASMD type B is characterized by an unpredictable age of onset, variable disease course, and absence of neurological complications. Hepatosplenomegaly is often the earliest clinical manifestation, and impaired pulmonary and liver functions may develop over time [7]. Thrombocytopenia is the most common hematologic manifestation associated with ASMD. Patients may experience bleeding manifestations, such as recurrent epistaxis, that occasionally require repeated cauterization [5]. Significant bleeding incidents have also been reported, including hemoptysis, hematemesis, excessive post-operative bleeding (following tonsillectomy or adenoidectomy), hemothorax, and subdural hematomas, which may require blood transfusions [8]. In female patients, menorrhagia and uterine bleeding could be severe enough to indicate hysterectomy [5]. Bleeding, disproportionate to the extent of the injury or surgery performed, and easy bruising are more frequently observed than anemia in these patients.

Various mechanisms may lead to thrombocytopenia in ASMD. Splenomegaly leads to sequestration of platelets and consequently diminishes their circulating levels [1]. Additionally, lipid accumulation within bone marrow macrophages interferes with megakaryocyte maturation and subsequent platelet release. The accumulation of sphingomyelin and cholesterol alters the platelet membrane composition, resulting in impaired aggregation and adhesion [9]. Furthermore, disrupted calcium signaling in the platelets of ASMD patients reduces platelet functionality [10].

In previous years, the absence of effective disease-modifying treatments limited the management of ASMD to symptomatic treatment and supportive care [8]. Olipudase alfa (Xenpozyme™) is a recombinant human acid sphingomyelinase (rhASM) that serves as an intravenous enzyme replacement therapy (ERT). It is approved for managing non-central nervous system manifestations of ASMD in both pediatric and adult patients [11, 12]. However, there is limited real-world data beyond the context of clinical trials, especially in the pediatric population. This study aims to investigate the efficacy and safety of olipudase alfa as ERT for hematological and visceral manifestations in children diagnosed with ASMD B or A/B.

## Patients and methods

This prospective interventional study was conducted at the Hematology Unit of Assiut University Children’s Hospital in Egypt. It included ten pediatric patients diagnosed with ASMD, confirmed by diminished acid sphingomyelinase enzyme activity and subsequently validated through genetic testing. The study was approved by the Ethics Committee of the Faculty of Medicine, Assiut University (Approval number: 04-300547), and informed consent was secured from the legal guardians of all participating patients.

### Acid Sphingomyelinase enzyme assay

Acid sphingomyelinase enzyme assay was measured using tandem mass spectrometry from dried blood spots. Enzyme deficiency was defined as an activity below the cutoff value of 1.2 µmol/L/h.

### Single-gene sequencing (ASMP1 gene)

DNA extraction and next-generation sequencing (NGS) were performed, covering all coding exons (including partial exon-2) and flanking intronic regions. The obtained sequences were aligned to the reference sequence NM_000543.4 (ENST00000342245) provided by Archimed Life International Medical Laboratory. Gene ID:6609 detected variants were described according to the Human Genome Variation Society (HGVS) recommendations for the description of sequence variants (Hum Mutat. 2016 Jun; 37(6):564-9) [13].

### Data collection

All enrolled patients underwent a thorough medical history assessment, including age at presentation, disease duration, history of bleeding, and relevant family history. All patients were subjected to a comprehensive clinical examination. Neurological evaluation was conducted by a pediatric neurologist, and the ASMD subtype was classified by the treating physicians. ASMD type B was defined by the absence of neurological signs throughout the disease course. ASMD type A/B (chronic neurovisceral variant) was determined by the age of onset and rate of progression of neurological manifestations. Patients with ASMD type A were excluded from the study.

Weight and height Z-scores were assessed every 12 weeks using World Health Organization (WHO) growth standards. Blood samples were collected at baseline and every three months for liver function tests, lipid profiles, and complete blood count (CBC). Liver and spleen sizes were measured in centimeters using standard abdominal ultrasonography, performed every three months by an experienced radiologist. Liver measurements were taken along the midclavicular line, extending from the dome to the inferior border. Spleen size was recorded by measuring the maximum longitudinal length (pole-to-pole). A baseline fundus examination was performed by a qualified pediatric ophthalmologist.

All patients were too young to undergo pulmonary function testing. High-resolution computed tomography (HRCT) of the chest was performed before and after treatment. A pediatric radiologist independently interpreted and scored the imaging results. A standardized 4-point scoring system was used to assess the presence of thickened interlobular septa, intralobular lines, ground glass opacities, and the overall extent of lung involvement. Scores were assigned as follows: 0 indicating no interstitial disease, 1 for 1–25% of lung volume affected, 2 for 26–50%, and 3 for 51–100% [14]. Bone mineral density was evaluated using dual energy X-ray absorptiometry (DXA), with age-adjusted Z scores calculated from lumbar spine images (L1-L4) obtained at baseline and one year after treatment [15].

### Treatment plan

All patients received olipudase alfa for 12 months, following a pediatric dose-escalation protocol every two weeks. Treatment commenced at a starting dose of 0.03 mg/kg and was gradually increased to a target dose of 3 mg/kg. Due to the previously reported high frequency of mild infusion-related reactions (IARs) in pediatric populations, all patients received premedication with intravenous antihistamines and corticosteroids prior to each infusion [16]. Olipudase alfa has been associated with transient elevations in transaminases during dose escalation. Therefore, alanine aminotransferase (ALT), aspartate aminotransferase (AST), total bilirubin, and direct bilirubin concentration were monitored prior to and 48 h after each infusion during this phase, as indicators of potential **hepatocellular** injury [11]. All treatment-related adverse events (AEs), including IAR (hypersensitivity or acute phase responses), as well as any liver function impairment, were documented and categorized as mild, moderate, or severe as per the olipudase alfa administration protocol. If moderate or severe AEs were observed, dose de-escalation or treatment discontinuation was implemented as per protocol [12].

### Statistical analysis

Data analysis was carried out using SPSS version 20. Results were expressed as mean ± standard error (SE). The Kolmogorov–Smirnov and Shapiro–Wilk tests were used to assess the normality of data distribution. Paired t-test was used to compare values before and after treatment in normally distributed values. Wilcoxon signed-rank test was applied for non-normally distributed data. A p-value of < 0.05 was considered statistically significant.

## Results

A total of ten children were enrolled in this prospective study, including two females and eight males, with a mean age of 3.1 ± 1.52 years (range: 2–6 years). Demographic and baseline clinical characteristics of the patients are summarized in Table 1. All ten patients (100%) presented with anemia, and six patients (60%) were found to have thrombocytopenia. Four patients (40%) experienced various bleeding episodes, including epistaxis, gingival bleeding, and ecchymosis. None of the children in the current cohort exhibited neutropenia. Splenomegaly was observed in all ten patients, while nine patients presented with hepatomegaly and dyslipidemia. Five children exhibited neurological symptoms (four with hypotonia or hyporeflexia, and one with cognitive decline and spasticity). These five patients were phenotypically classified as ASMD type A/B, and two of them were twins (patients 6 and 7).

**Table 1:** Baseline demographics and clinical characteristics of children with ASMD

Variable	Patient 1	Patient 2	Patient 3	Patient 4	Patient 5	Patient 6	Patient 7	Patient 8	Patient 9	Patient 10	Mean ± SD
Age (years)	3	2	2.3	2	6	4.2	4	2.3	4.5	3	3.3 ± 1.32
Gender	M	M	M	M	M	M	F	F	M	M	
Height Z-score	-1.47	-2.76	-4.22	-1.21	0.3	-6.8	-6.77	-3.07	-1.07	-3.39	-3.05 ± 2.37
Weight Z-score	-3.75	-3.08	-3.36	-4.46	-1.44	-4.8	-3.98	-4.59	0.62	-0.61	-2.94 ± 1.85
Hemoglobin (g/dL)	9	8.7	8.3	9.9	10.3	9.3	8.6	9.3	9.8	10.2	9.46 ± 0.87
ANC	2134	1789	2330	1987	3908	2987	3459	2734	3256	1632	2621.6 ± 767.5
Platelet Count (10^9/L)	97	78	167	89	45	67	156	66	211	181	95.62 ± 43.68
Bleeding	No	Yes	No	No	Yes	Yes	No	Yes	No	No	
AST (U/L)	221	345	403	90	19	119	432	309	118	78	235 ± 140.37
ALT (U/L)	182	187	302	66	39	86	316	213	151	56	173.22 ± 94.97
HDL (mg/dL)	26	25	18.4	19.6	25.4	29	25	28	30	24	25.04 ± 3.72
LDL (mg/dL)	187	149	153.1	134.2	96	127	220	167.4	184	112.3	153± 37.7
Cholesterol (mg/dL)	232	231	287	234	145	243	287	259	216	204	243.67 ± 28.98
Triglycerides (mg/dL)	245	216	342	254	117.7	341	213	268	197	189	238.27 ± 68.55
Pulmonary Involvement	,		Yes						Yes		
Liver Size (cm)	14.3	14	16.7	13.4	11.3	12.7	14	14.2	15.5	14	14.01 ± 1.45
Spleen Size (cm)	14.9	12	15.3	12.8	17.8	15	12	12.8	11.7	12.4	13.67 ± 1.98
DXA Z-score	-3.7	-4.9	-5.7	-3.8	-2.2	-4.1	-4.6	-5.1	-2.4	-3.6	-4.01 ± 1.12
Fundus	N	Cherry red spot	N	N	N	N	N	Cherry red spot	N	N	

N: Normal; WT: weight; ANC: Absolute neutrophilic count; Plt: Platelets; ALT: Alanine aminotransferase; AST: Aspartate aminotransferase; PH: pulmonary hypertension; Pulm: Pulmonary involvement; HDL: high-density lipoprotein; LDL: low density lipoprotein; Chol: total cholesterol; TG: triglyceride; DXA: dual energy X-ray absorptiometry

The remaining five patients were classified as ASMD type B. Four of them exhibited the *p.(Arg610del)* neuroprotective gene mutation in a heterozygous state (Table 2). Cherry red spots were observed in two patients, both of whom were classified as ASMD type A/B. Two patients exhibited pulmonary involvement. Patient 3 had an interstitial lung disease (ILD) score of 2. Patient 9 presented with an ILD score of 3, with mild pulmonary hypertension and required supplemental oxygen.

**Table 2:** Genotype/Phenotype correlation

Patient	Gene Variant (cDNA)	Protein Change	Zygosity	Neurological Manifestations	Phenotype
1	c.1406 A > C / c.1406 A > C	p.(Trp469Ser)	Homozygous	No	B
2	c.1381 C > G / c.1148 A > G	p.(His461Asp) / p.(Asn383Ser)	Compound Heterozygous	Yes	A/B
3	c.430G > A / c.1148 A > G	p.(Val144Met) / p.(Asn383Ser)	Compound Heterozygous	Yes	A/B
4	c.1624 C > T / c.1829_1831del	p.(Arg542Ter) / p.(Arg610del)	Compound Heterozygous	No	B
5	c.1829_1831del / c.1829_1831del	p.(Arg610del)	Homozygous	No	B
6	c.1148 A > G / c.1148 A > G	p.(Asn383Ser)	Homozygous	Yes	A/B
7	c.1148 A > G / c.1148 A > G	p.(Asn383Ser)	Homozygous	Yes	A/B
8	c.682T > G / c.1406 A > C	p.(Cys228Gly) / p.(Trp469Ser)	Compound Heterozygous	Yes	A/B
9	c.682T > G / c.1829_1831del	p.(Cys228Gly) / p.(Arg610del)	Compound Heterozygous	No	B
10	c.1381 C > G / c.1829_1831del	p.(His461Asp) / p.(Arg610del)	Compound Heterozygous	No	B

### Safety and adverse events during dose escalation

The olipudase alfa dose was successfully escalated to the target dose of 3 mg/kg in all patients. No patients suffered from severe IRA that necessitated dose modifications. Only one patient experienced a mild urticarial rash at week 4 (during step two of the escalation protocol), and another patient developed pyrexia. Transient elevations in transaminase concentrations were observed in three patients during the dose escalation phase. Patient 3 experienced two episodes of elevated transaminases at doses of 0.3 mg and 1 mg. AST concentration reached 469 U/L and 444 U/L, respectively (reference range: 10–40 U/L), and ALT concentrations reached 354 U/L and 253 U/L, respectively (reference range: ≤50 U/L). Patient 9 exhibited a significant elevation at olipudase alfa dosage of 0.1 mg, with AST and ALT concentrations reaching 167 U/L, and 172 U/L, respectively. Both patients remained asymptomatic and transaminase concentrations normalized before the subsequent scheduled injection. Patient 2 experienced a single episode of asymptomatic transaminase elevation while receiving a dosage of 0.3 mg/kg, where AST and ALT concentrations reached 545 U/L and 356 U/L, respectively. The same dosage was re-administered during the subsequent infusion without any further complications, and dose escalation continued as planned.

### One-year outcomes

After one year of olipudase alfa treatment, a significant improvement in weight Z-scores was observed in all children compared to baseline measurements (*p* = 0.049). However, height Z-scores showed no statistically significant change (Table 3).

**Table 3:** Clinical and laboratory parameters before and after treatment

	Before Treatment (*n* = 10)	After Treatment (*n* = 10)	*P*-value
Weight Z-score	-2.94 ± 1.85	-1.2 ± 2.43	0.049
Height Z-score	-3.05 ± 23.7 2.37	-3.13 ± 1.78	0. 909
Hb (g/dl)	9.46 ± 0.87	11.35 ± 0.783	0 0.0001
Platelets (10^9^/L) (*n* = 6)^&^	95.62 ± 43.68	214.17 ± 75.93	0.0057
Liver (cm)*	14.01 ± 1.45	11.08 ± 0.94	0.0012
Spleen (cm)	13.67 ± 1.98	10.6 ± 0.94	0.00041
AST (U/L)*	235 ± 140.37	39.06 ± 36.9	0.0045
ALT (U/L)*	173.22 ± 94.97	43.91 ± 19.5	0.0019
Total cholesterol (mg/dL)* (Normal range: ≤200 mg/dL)	243.67 ± 28.98	172.83 ± 34.5	0.0029
Triglyceride (mg/dL)* (Men 40–160 mg/dL)	238.27 ± 68.55	131.26 ± 45.8	0.008
HDL (mg/dL) (Normal range: 35–60 mg/dL)	25.04 ± 3.72	34.28 ± 7.14	0.023
DXA Z-score	-4.01 ± 1.12	-3.42 ± 0.95	0.008

*Patient 9; Hb: Hemoglobin; ALT: Alanine aminotransferase; AST: Aspartate aminotransferase; HDL: high-density lipoprotein; DXA; dual energy X-ray absorptiometry

### Hematologic outcomes

**Fig. 1:**
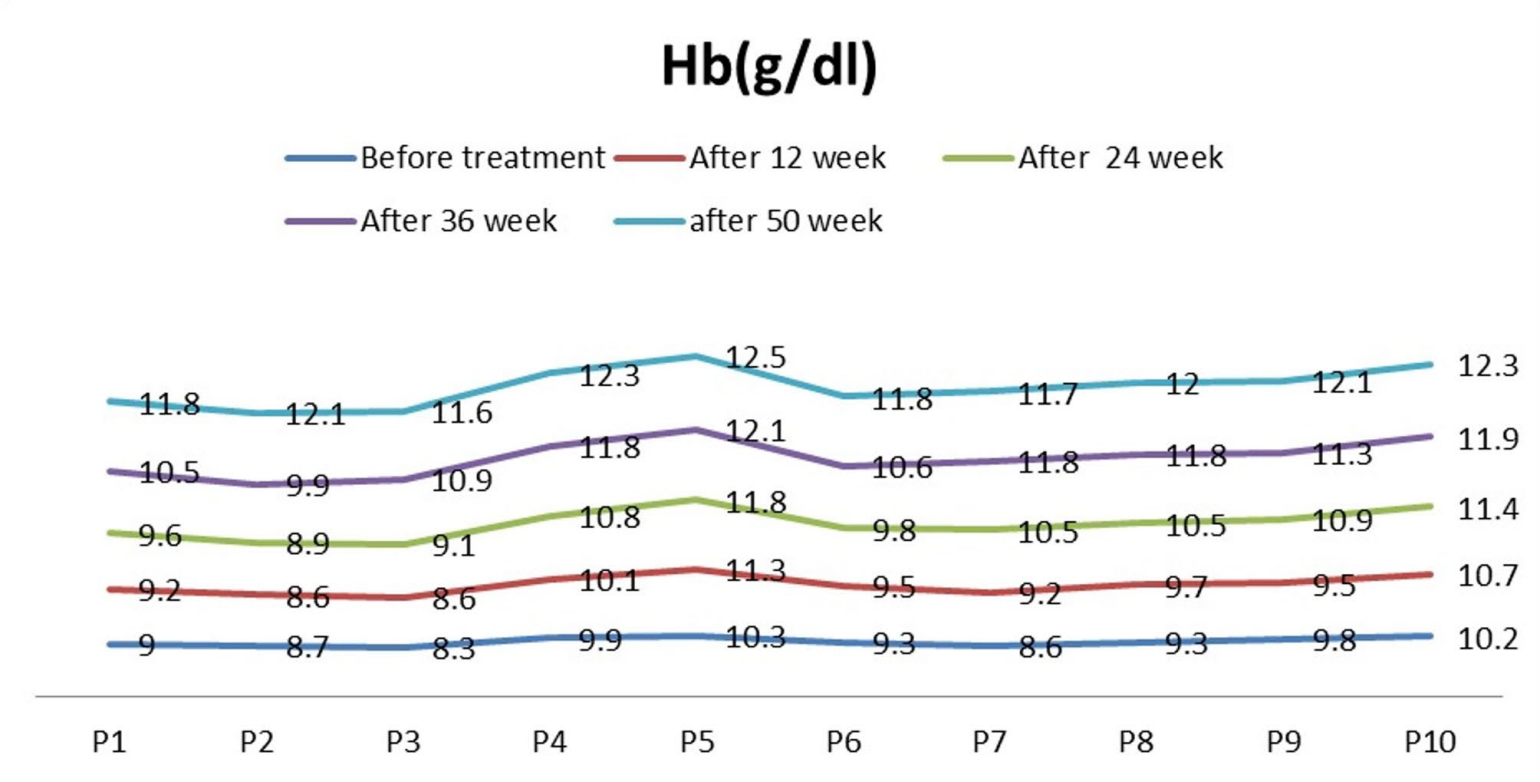
Hemoglobin levels (Hb, g/dL) from baseline to week 50 during olipudase alfa treatment. Significant increases were observed at week 12 (*p* = 0.02), week 24 (*p* = 0.001), week 36 (*p* = 0.0001), and week 50 (*p* < 0.0001)

**Fig. 2:**
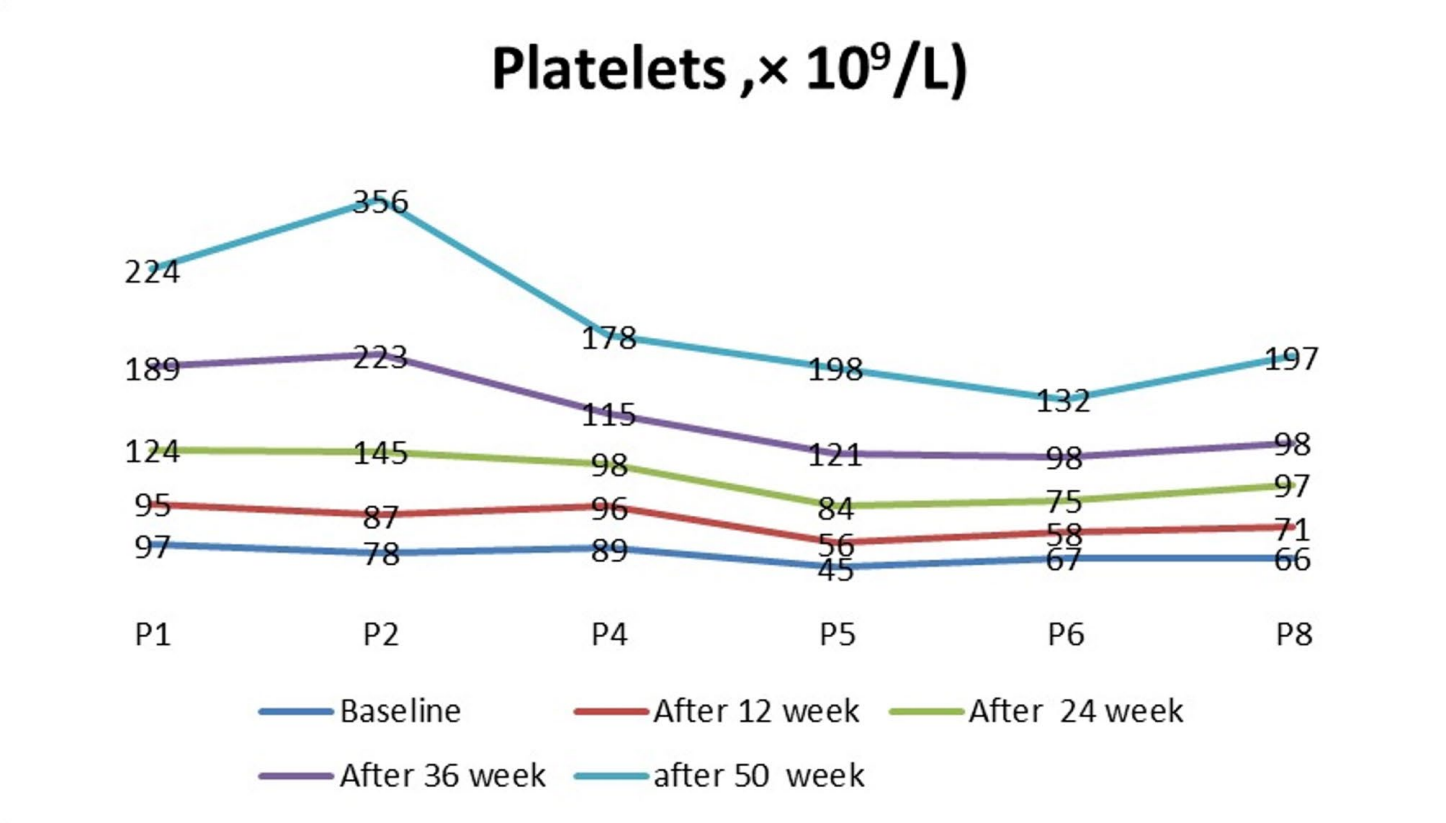
Platelet counts from baseline to week 50 during olipudase alfa treatment. No significant change at week 12 (*p* = 0.3), followed by significant increases at week 24 (*p* = 0.0196), week 36 (*p* = 0.017), and week 50 (*p* = 0.0057). Seven patients achieved normal platelet counts after 48 weeks of treatment

Hematologically, hemoglobin levels showed a significant increase starting at week 12 of treatment (*p* = 0.02), with a gradual increase reaching peak levels at week 50 (*p* < 0.0001), as illustrated in Fig. 1. In enrolled ASMD patients with thrombocytopenia, platelet counts did not exhibit a significant change at week 12 of treatment (*p* = 0.3). However, a significant increase in platelet counts was observed at week 24 (*p* = 0.0196), with a gradual increase reaching peak levels at week 50 (*p* = 0.0057). By the end of the study, five patients had achieved platelet counts within the normal range, as shown in Fig. 2.

### Transaminase outcomes

Elevated transaminase concentrations were observed in nine patients at baseline. Eight children demonstrated a gradual decline in transaminase concentrations starting at week 16 and reached the normal reference range by week 40. Specifically, AST concentration significantly decreased from 235 ± 140.37 U/L to 39.06 ± 36.9 U/L (*p* = 0.0045), while ALT concentration dropped from 173.22 ± 94.97 U/L to 43.91 ± 19.5 U/L (*p* = 0.0019) (Table 3). However, Patient 8 exhibited a different pattern, as their transaminase concentrations started to decrease at week 20 and ultimately returned to the normal reference range by week 46.

### Lipid profile outcomes

All patients presented at baseline with dyslipidemia, characterized by elevated total cholesterol and triglycerides (TG) levels, or a reduced high-density lipoprotein cholesterol (HDL-C) level [17]. Following olipudase alfa treatment, there was a significant reduction in cholesterol and TG levels, falling within normal reference ranges by week 40 (*p* = 0.0029 and *p* = 0.008, respectively). HDL-C levels showed a significant increase (*p* = 0.023); however, levels remained below the lower limit of the normal reference range (Table 3).

### Pulmonary and skeletal outcomes

Both patients with pulmonary involvement demonstrated a marked improvement in ILD, with a notable decrease in reticular granularity, as assessed by HRCT. ILD scores decreased from 2 to 1.225 in Patient 3 and from 3 to 0.975 in Patient 9, who subsequently became independent of supplemental oxygen. In addition, both patients showed a reduction in the frequency of hospital admissions and reported no respiratory symptoms during the final three months of follow-up. All patients had no history of pathological fractures. Bone mineral density in the lumbar spine (L1-L4) showed a significant increase in age-matched Z-score from a baseline mean of -4.01 ± 1.12 to -3.42 ± 0.95 after one year (*p* = 0.008).

## Discussion

In recent years, the treatment landscape of ASMD has evolved significantly. Previously, no disease-specific medications were available; however, the introduction of ERT with recombinant human acid sphingomyelinase, olipudase alfa, marked a major advancement in therapeutic options for this disease [18].

In the current study, five patients were diagnosed with the chronic neurovisceral form of ASMD (ASMD type A/B). These patients exhibited early neurological symptoms; however, no significant neurodegeneration was observed during follow-up evaluations. Furthermore, none of them carried alleles typically associated with the infantile neurovisceral phenotype [19]. Based on their genotypes and clinical features, they were diagnosed with chronic neurovisceral ASMD and deemed suitable for ERT treatment. Determining whether to initiate ERT in ASMD patients with early neurological manifestations can be challenging, especially during early childhood. Distinguishing between chronic neurovisceral ASMD and infantile neurovisceral ASMD can be difficult. Consequently, physicians should continuously monitor disease progression and reassess diagnosis if the patient’s condition continues to deteriorate despite ERT.

The remaining five patients were diagnosed with the chronic visceral form of ASMD. Four patients possessed the pathogenic *p.Arg610del* variant, which is linked to the absence of neurodegenerative features in patients who are either homozygous or compound heterozygous [20]. Currently, there is a scarcity of published data on the use of olipudase alfa in pediatric patients, particularly outside the clinical trial setting.

To the best of our knowledge, this study represents the largest case series of pediatric patients with ASMD, providing insights into the real-world application of olipudase alfa in this population. This study builds on the study by ***Yu-Wen Pan et al.***, who previously reported the safety and efficacy of ERT in only two children diagnosed with the chronic neurovisceral form of ASMD (type A/B) [21]. All enrolled children tolerated olipudase alfa therapy well, with no significant adverse events reported requiring dose adjustments. Only one patient developed a mild urticarial rash, and another developed pyrexia, with no required changes in the treatment regimen. The ASCEND-PED study, an international, multicenter, non-randomized phase 1 and 2 clinical trial, evaluated the safety and efficacy of olipudase alfa in ASMD patients under 18 years of age. Twenty participants were included in the study: four adolescents (12–17 years), nine children (6–11 years), and seven infants and young children (1–5 years). The majority of adverse events were considered mild or moderate in severity. Infusion-related reactions, primarily manifesting as urticaria, fever, and/or vomiting, were reported in eleven patients. It is noteworthy that none of our patients experienced anaphylaxis, in contrast to the ASCEND-PED study, which reported a case of anaphylaxis associated with anti-drug antibodies (ADA+) that required desensitization [16].

In the present study, anemia was identified in all participating children (100%). In contrast, a previous prospective cross-sectional survey reported anemia in only 26% of the 59 patients evaluated [22]. The higher prevalence observed in our study could be attributed to the younger average age of our participants (mean age: 3.33 ± 1.32 years) compared to the survey’s population (mean age: 22.6 ± 13.8 years). A notable hematologic finding in our cohort was the substantial increase in hemoglobin levels following 12 weeks of consistent treatment. Hemoglobin levels continued to improve, ultimately reaching peak levels at week 50. Mild to moderate thrombocytopenia was detected in 60% of our patients, with five patients (50%) experiencing bleeding episodes, most commonly epistaxis. Previous studies have also documented thrombocytopenia in patients with ASMD. A cross-sectional study reported thrombocytopenia in 53% of the ASMD patients at the time of evaluation, establishing it as the most common hematologic abnormality. Additionally, the study reported bleeding episodes in 49% of those patients, with recurrent epistaxis reported in 29% of the participants [22]. Thrombocytopenia was reported in 25% of patients in the ASCEND-PED study, while 10% experienced significant bleeding and bruising [15].

In our cohort, a significant increase in platelet count was observed after 24 weeks of olipudase alfa treatment. It continued to rise until five patients reached the normal range by week 50. A comparable finding was reported in the ASCEND-PED study, where platelet counts increased by 31.76 ± 8.09% at week 52 in the infant/early childhood group (*p* = 0.0172) [15]. One patient in our study showed a significant increase in platelet count, yet remained within the low-normal range, a finding consistent with the study by Yu-Wen Pan et al. [21].

A significant clinical outcome observed in our cohort was the reduction in the average size of both the liver (in 9 out of 10 patients) and spleen, *p* = 0.00012 and *p* = 0.00041, respectively. This finding is consistent with results from the ASCEND-PED study, which reported more than a 40% reduction in mean liver and spleen sizes (*p* < 0.0001) [12]. Similarly, Yu-Wen Pan and colleagues documented reductions in hepatic and splenic volumes, along with decreased liver stiffness [21]. The observed decrease in splenomegaly in our study was associated with an increase in platelet counts across all pediatric groups, suggesting resolution of secondary hypersplenism.

Elevated transaminases and dyslipidemia were detected in 90% of our patients at baseline. By week 40, transaminase concentrations, total cholesterol, and triglycerides significantly decreased to the normal reference range in all nine patients, except for one patient who achieved these reductions by week 46. Although HDL levels increased significantly in all patients, they remained below the lower normal limit. Similar findings were reported in the study conducted by Yu-Wen Pan et al. [21], while in the ASCEND-PED trial, although all patients achieved normal mean lipid concentrations by week 52, two patients continued to exhibit elevated transaminase concentrations [16].

In the current study, two patients exhibited significant pulmonary involvement, with one patient being dependent on supplemental oxygen. However, both patients were ineligible for pulmonary function testing due to their young ages. After one year of ERT, both patients exhibited a substantial radiological improvement, with marked reductions in reticular granularity and ILD, as demonstrated by HRCT. ILD scores decreased from 2 to 1.225 in Patient 3, and from 3 to 0.975 in Patient 9, who subsequently achieved independence from supplemental oxygen. These findings align with the results reported by Yu-Wen Pan et al. [21], who observed a reduction in ILD scores from 3 to 0.625 in Patient 1 and from 2.5 to 1.375 in Patient 2, after one year of treatment. Additionally, the ASCEND-PED study10 revealed a reduction in baseline mean ILD scores from 2.6 ± 0.6 and 2.5 ± 0.8 in the child and infant/early child groups, respectively, to 2.0 ± 1.1 and 1.9 ± 1.3 at week 52. Concerning the pattern of growth, all our patients exhibited failure to thrive at baseline. After one year of treatment with olipudase alfa, a notable increase in weight z-score was observed among all children. However, there was no significant change in the height z-score. Yu-Wen Pan and colleagues [21] reported an enhancement in height Z scores at the 50th week following treatment. In contrast, the ASCEND-PED study observed that by week 52, 79% of patients (15 out of 19) showed improvement in height Z-scores, with the remaining 21% exhibiting no change [16]. Furthermore, the age-matched Z-scores for bone mineral density in the lumbar spine (L1-L4) showed significant improvement, consistent with a previous study [21].

After one year of treatment, we did not observe any significant improvements or deterioration in neurological manifestations or neurocognitive function, as assessed by a pediatric neurologist. Olipudase alfa does not penetrate the blood-brain barrier; consequently, it lacks clinical efficacy in treating central nervous system involvement [23]. Therefore, olipudase alfa’s therapeutic indication is limited to addressing non-CNS manifestations of ASMD in both pediatric and adult patients [8, 22].

### Limitations

This study had several limitations. Firstly, abdominal computed tomography (CT) or magnetic resonance imaging (MRI) were not used to assess liver volume to avoid the risks associated with sedation in young children. Furthermore, we did not evaluate liver stiffness using ultrasound elastography. Secondly, lung function testing was not conducted as our patients were too young to cooperate with the procedure. Lastly, the study was limited by a small sample size. However, ASMD is classified as an ultra-rare genetic disorder.

## Conclusion

Our findings, consistent with previously published studies, indicate significant clinical benefits of olipudase alfa in pediatric patients with ASMD. The treatment is well-tolerated and effective, resulting in marked improvements in hematological parameters, hepatosplenomegaly, bone mineral density, pulmonary involvement, and dyslipidemia. A longer-term follow-up period will be planned to assess the ongoing benefits of enzyme replacement therapy in chronic ASMD patients.

## Data Availability

No datasets were generated or analysed during the current study.

## Abbreviations

ADA: Anti-drug antibodies
ALT: Alanine aminotransferase
ASMD: Acid sphingomyelinase deficiency
AST: Aspartate aminotransferase
CBC: Complete blood count
CT: Computed tomography
DXA: Dual energy X-ray absorptiometry
ERT: Enzyme replacement therapy
HDL-C: High-density lipoprotein cholesterol
HGVS: Human Genome Variation Society
HRCT: High-resolution computed tomography
IAR: Infusion-related reactions
ILD: Interstitial lung disease
LDL: Low-density lipoprotein
MRI: Magnetic resonance imaging
SE: Standard error
